# Supplementary material for: Site-saturation mutagenesis of 500 human protein domains
Source: Nature. 2025 Jan 8;637(8047):885–94. doi: 10.1038/s41586-024-08370-4 (PMC11754108; doi:10.1038/s41586-024-08370-4)
Supplement: Supplementary file 1 — This file contains Supplementary Fig. 1 and Supplementary Tables 6–8. [file 41586_2024_8370_MOESM1_ESM.pdf]

nature portfolio

https://doi.org/10.1038/s41586-024-08370-4

Supplementary information

Site-saturation mutagenesis of 500 human protein domains

In the format provided by the authors and unedited

Supplementary Figures

Supplementary Figure 1: Deep mutational scans of protein abundance of 522 protein domains. Domains are sorted by protein family and ranked by quality within families.

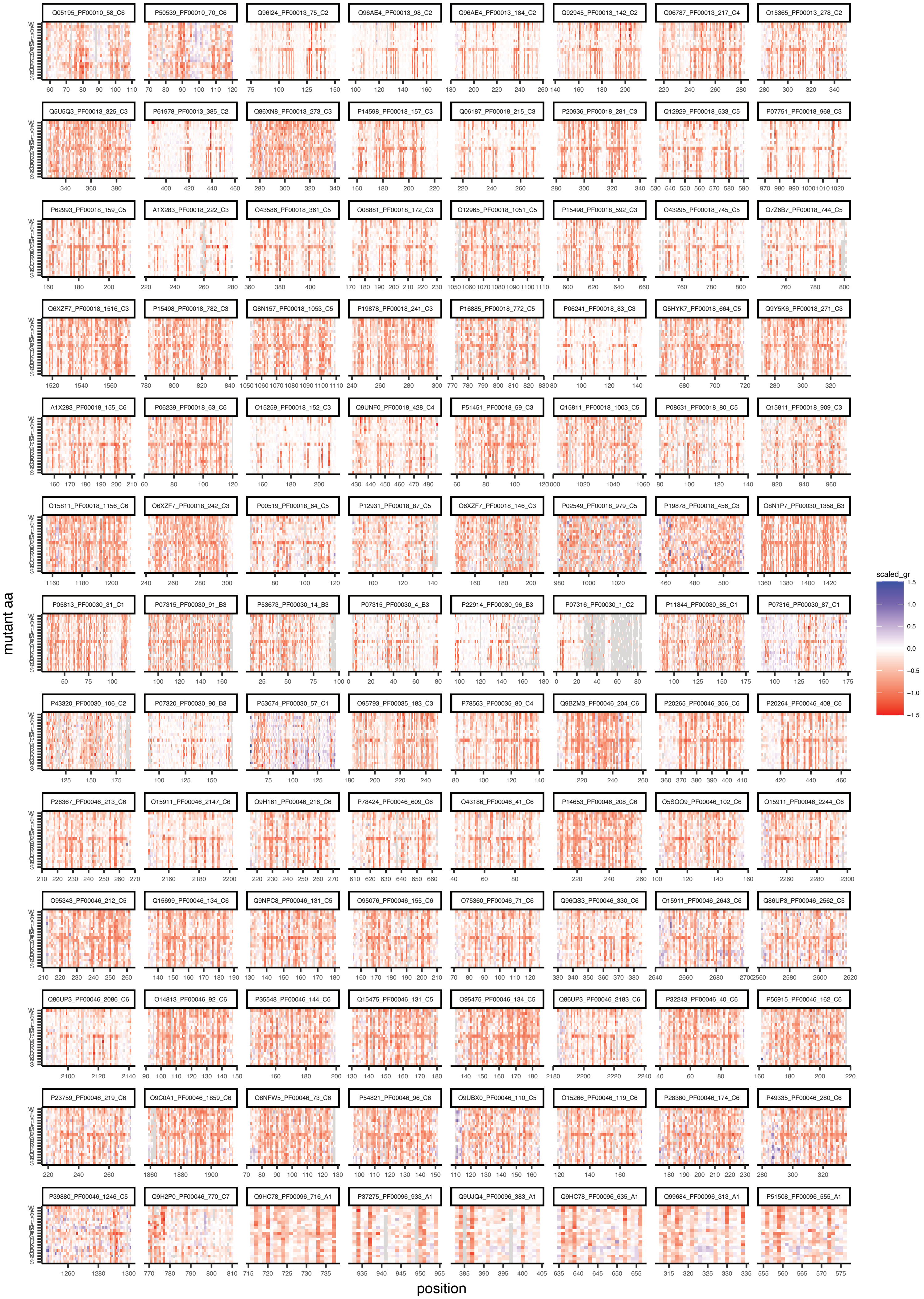

scaled_gr
mutant aa
position
Q05195_PF00010_58_C6
P50539_PF00010_70_C6
Q96I24_PF00013_75_C2
Q96AE4_PF00013_98_C2
Q96AE4_PF00013_184_C2
Q92945_PF00013_142_C2
Q06787_PF00013_217_C4
Q15365_PF00013_278_C2
Q5U5Q3_PF00013_325_C3
P61978_PF00013_385_C2
Q86XN8_PF00013_273_C3
P14598_PF00018_157_C3
Q06187_PF00018_215_C3
P20936_PF00018_281_C3
Q12929_PF00018_533_C5
P07751_PF00018_968_C3
P62993_PF00018_159_C5
A1X283_PF00018_222_C3
O43586_PF00018_361_C5
Q08881_PF00018_172_C3
Q12965_PF00018_1051_C5
P15498_PF00018_592_C3
O43295_PF00018_745_C5
Q7Z6B7_PF00018_744_C5
Q6XZF7_PF00018_1516_C3
P15498_PF00018_782_C3
Q8N157_PF00018_1053_C5
P19878_PF00018_241_C3
P16885_PF00018_772_C5
P06241_PF00018_83_C3
Q5HYK7_PF00018_664_C5
Q9Y5K6_PF00018_271_C3
A1X283_PF00018_155_C6
P06239_PF00018_63_C6
O15259_PF00018_152_C3
Q9UNF0_PF00018_428_C4
P51451_PF00018_59_C3
Q15811_PF00018_1003_C5
P08631_PF00018_80_C5
Q15811_PF00018_909_C3
Q15811_PF00018_1156_C6
Q6XZF7_PF00018_242_C3
P00519_PF00018_64_C5
P12931_PF00018_87_C5
Q6XZF7_PF00018_146_C3
P02549_PF00018_979_C5
P19878_PF00018_456_C3
Q8N1P7_PF00030_1358_B3
P05813_PF00030_31_C1
P07315_PF00030_91_B3
P53673_PF00030_14_B3
P07315_PF00030_4_B3
P22914_PF00030_96_B3
P07316_PF00030_1_C2
P11844_PF00030_85_C1
P07316_PF00030_87_C1
P43320_PF00030_106_C2
P07320_PF00030_90_B3
P53674_PF00030_57_C1
O95793_PF00035_183_C3
P78563_PF00035_80_C4
Q9BZM3_PF00046_204_C6
P20265_PF00046_356_C6
P20264_PF00046_408_C6
P26367_PF00046_213_C6
Q15911_PF00046_2147_C6
Q9H161_PF00046_216_C6
P78424_PF00046_609_C6
O43186_PF00046_41_C6
P14653_PF00046_208_C6
Q5SQQ9_PF00046_102_C6
Q15911_PF00046_2244_C6
O95343_PF00046_212_C5
Q15699_PF00046_134_C6
Q9NPC8_PF00046_131_C5
O95076_PF00046_155_C6
O75360_PF00046_71_C6
Q96QS3_PF00046_330_C6
Q15911_PF00046_2643_C6
Q86UP3_PF00046_2562_C5
Q86UP3_PF00046_2086_C6
O14813_PF00046_92_C6
P35548_PF00046_144_C6
Q15475_PF00046_131_C5
O95475_PF00046_134_C5
Q86UP3_PF00046_2183_C6
P32243_PF00046_40_C6
P56915_PF00046_162_C6
P23759_PF00046_219_C6
Q9C0A1_PF00046_1859_C6
Q8NFW5_PF00046_73_C6
P54821_PF00046_96_C6
Q9UBX0_PF00046_110_C5
O15266_PF00046_119_C6
P28360_PF00046_174_C6
P49335_PF00046_280_C6
P39880_PF00046_1246_C5
Q9H2P0_PF00046_770_C7
Q9HC78_PF00096_716_A1
P37275_PF00096_933_A1
Q9UJQ4_PF00096_383_A1
Q9HC78_PF00096_635_A1
Q99684_PF00096_313_A1
P51508_PF00096_555_A1

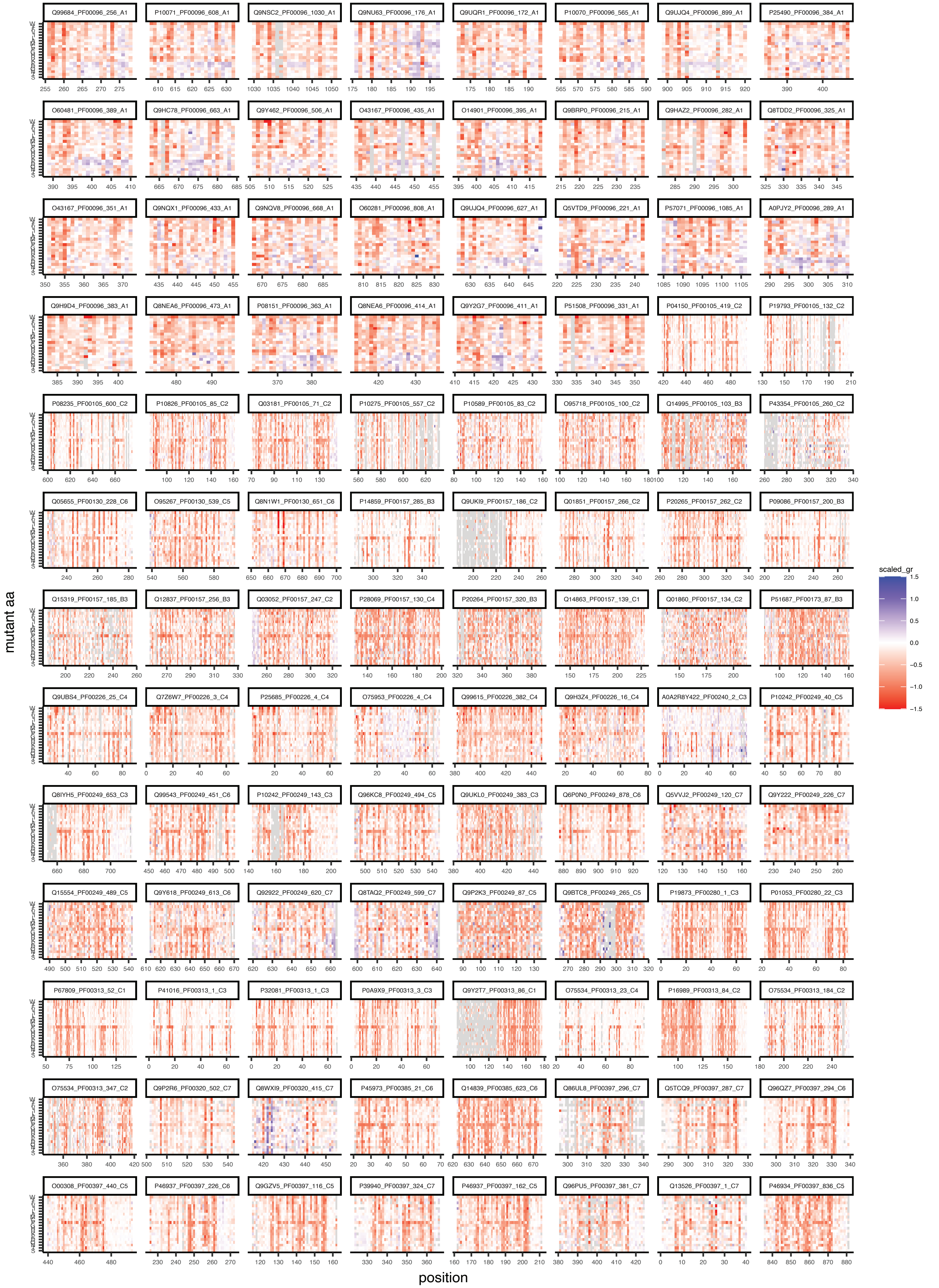
Q99684_PF00096_256_A1
P10071_PF00096_608_A1
Q9NSC2_PF00096_1030_A1
Q9NU63_PF00096_176_A1
Q9UQR1_PF00096_172_A1
P10070_PF00096_565_A1
Q9UJQ4_PF00096_899_A1
P25490_PF00096_384_A1
O60481_PF00096_389_A1
Q9HC78_PF00096_663_A1
Q9Y462_PF00096_506_A1
O43167_PF00096_435_A1
O14901_PF00096_395_A1
Q9BRP0_PF00096_215_A1
Q9HAZ2_PF00096_282_A1
Q8TDD2_PF00096_325_A1
O43167_PF00096_351_A1
Q9NQX1_PF00096_433_A1
Q9NQV8_PF00096_668_A1
O60281_PF00096_808_A1
Q9UJQ4_PF00096_627_A1
Q5VTD9_PF00096_221_A1
P57071_PF00096_1085_A1
A0PJY2_PF00096_289_A1
Q9H9D4_PF00096_383_A1
Q8NEA6_PF00096_473_A1
P08151_PF00096_363_A1
Q8NEA6_PF00096_414_A1
Q9Y2G7_PF00096_411_A1
P51508_PF00096_331_A1
P04150_PF00105_419_C2
P19793_PF00105_132_C2
P08235_PF00105_600_C2
P10826_PF00105_85_C2
Q03181_PF00105_71_C2
P10275_PF00105_557_C2
P10589_PF00105_83_C2
O95718_PF00105_100_C2
Q14995_PF00105_103_B3
P43354_PF00105_260_C2
Q05655_PF00130_228_C6
O95267_PF00130_539_C5
Q8N1W1_PF00130_651_C6
P14859_PF00157_285_B3
Q9UKI9_PF00157_186_C2
Q01851_PF00157_266_C2
P20265_PF00157_262_C2
P09086_PF00157_200_B3
Q15319_PF00157_185_B3
Q12837_PF00157_256_B3
Q03052_PF00157_247_C2
P28069_PF00157_130_C4
P20264_PF00157_320_B3
Q14863_PF00157_139_C1
Q01860_PF00157_134_C2
P51687_PF00173_87_B3
Q9UBS4_PF00226_25_C4
Q7Z6W7_PF00226_3_C4
P25685_PF00226_4_C4
O75953_PF00226_4_C4
Q99615_PF00226_382_C4
Q9H3Z4_PF00226_16_C4
A0A2R8Y422_PF00240_2_C3
P10242_PF00249_40_C5
Q8IYH5_PF00249_653_C3
Q99543_PF00249_451_C6
P10242_PF00249_143_C3
Q96KC8_PF00249_494_C5
Q9UKL0_PF00249_383_C3
Q6P0N0_PF00249_878_C6
Q5VVJ2_PF00249_120_C7
Q9Y222_PF00249_226_C7
Q15554_PF00249_489_C5
Q9Y618_PF00249_613_C6
Q92922_PF00249_620_C7
Q8TAQ2_PF00249_599_C7
Q9P2K3_PF00249_87_C5
Q9BTC8_PF00249_265_C5
P19873_PF00280_1_C3
P01053_PF00280_22_C3
P67809_PF00313_52_C1
P41016_PF00313_1_C3
P32081_PF00313_1_C3
P0A9X9_PF00313_3_C3
Q9Y2T7_PF00313_86_C1
O75534_PF00313_23_C4
P16989_PF00313_84_C2
O75534_PF00313_184_C2
O75534_PF00313_347_C2
Q9P2R6_PF00320_502_C7
Q8WXI9_PF00320_415_C7
P45973_PF00385_21_C6
Q14839_PF00385_623_C6
Q86UL8_PF00397_296_C7
Q5TCQ9_PF00397_287_C7
Q96QZ7_PF00397_294_C6
O00308_PF00397_440_C5
P46937_PF00397_226_C6
Q9GZV5_PF00397_116_C5
P39940_PF00397_324_C7
P46937_PF00397_162_C5
Q96PU5_PF00397_381_C7
Q13526_PF00397_1_C7
P46934_PF00397_836_C5
mutant aa
position
scaled_gr
1.5
1.0
0.5
0.0
−0.5
−1.0
−1.5

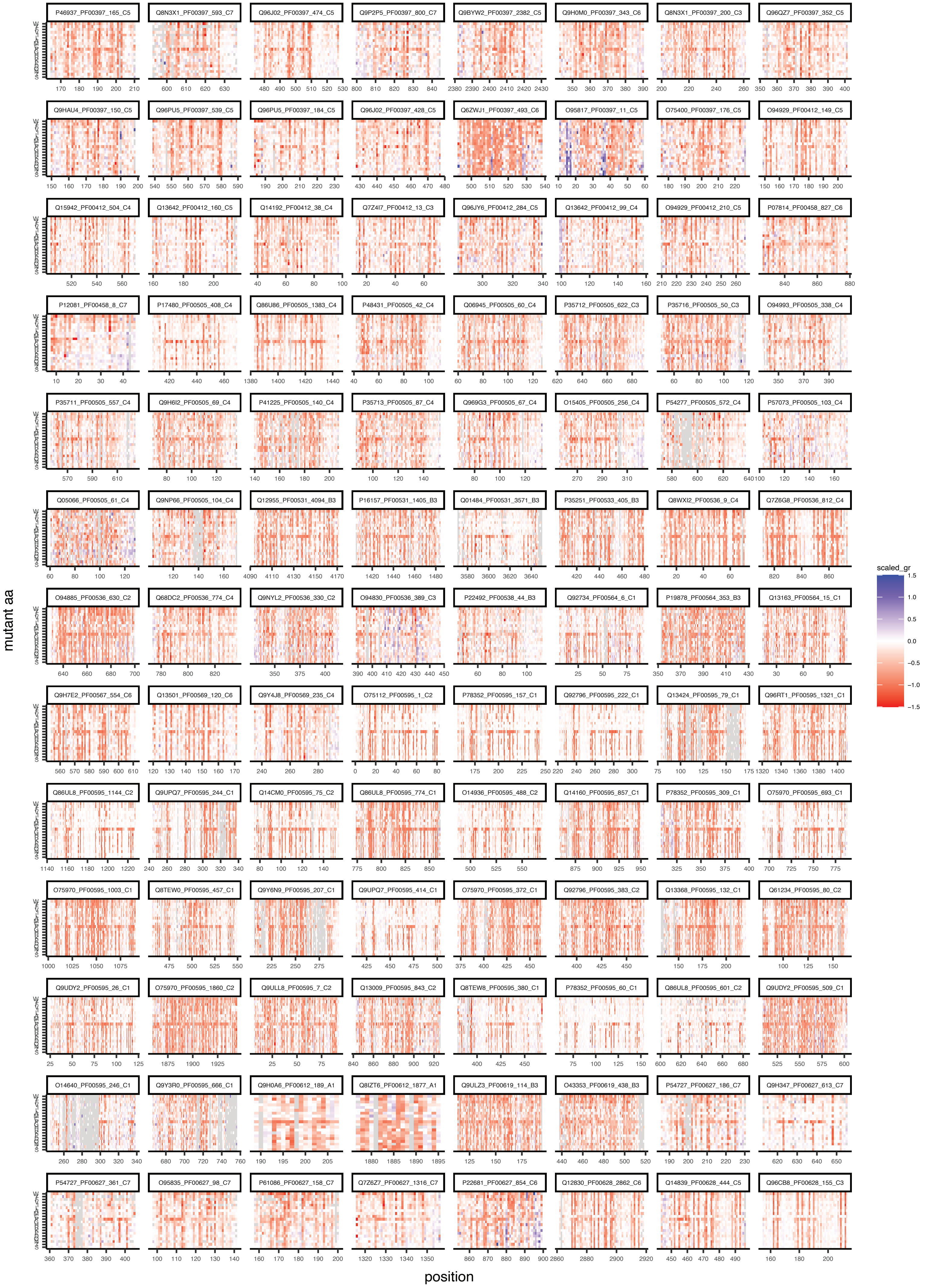
P46937_PF00397_165_C5
Q8N3X1_PF00397_593_C7
Q96J02_PF00397_474_C5
Q9P2P5_PF00397_800_C7
Q9BYW2_PF00397_2382_C5
Q9H0M0_PF00397_343_C6
Q8N3X1_PF00397_200_C3
Q96QZ7_PF00397_352_C5
Q9HAU4_PF00397_150_C5
Q96PU5_PF00397_539_C5
Q96PU5_PF00397_184_C5
Q96J02_PF00397_428_C5
Q6ZWJ1_PF00397_493_C6
O95817_PF00397_11_C5
O75400_PF00397_176_C5
O94929_PF00412_149_C5
Q15942_PF00412_504_C4
Q13642_PF00412_160_C5
Q14192_PF00412_38_C4
Q7Z4I7_PF00412_13_C3
Q96JY6_PF00412_284_C5
Q13642_PF00412_99_C4
O94929_PF00412_210_C5
P07814_PF00458_827_C6
P12081_PF00458_8_C7
P17480_PF00505_408_C4
Q86U86_PF00505_1383_C4
P48431_PF00505_42_C4
Q06945_PF00505_60_C4
P35712_PF00505_622_C3
P35716_PF00505_50_C3
O94993_PF00505_338_C4
P35711_PF00505_557_C4
Q9H6I2_PF00505_69_C4
P41225_PF00505_140_C4
P35713_PF00505_87_C4
Q969G3_PF00505_67_C4
O15405_PF00505_256_C4
P54277_PF00505_572_C4
P57073_PF00505_103_C4
Q05066_PF00505_61_C4
Q9NP66_PF00505_104_C4
Q12955_PF00531_4094_B3
P16157_PF00531_1405_B3
Q01484_PF00531_3571_B3
P35251_PF00533_405_B3
Q8WXI2_PF00536_9_C4
Q7Z6G8_PF00536_812_C4
O94885_PF00536_630_C2
Q68DC2_PF00536_774_C4
Q9NYL2_PF00536_330_C2
O94830_PF00536_389_C3
P22492_PF00538_44_B3
Q92734_PF00564_6_C1
P19878_PF00564_353_B3
Q13163_PF00564_15_C1
Q9H7E2_PF00567_554_C6
Q13501_PF00569_120_C6
Q9Y4J8_PF00569_235_C4
O75112_PF00595_1_C2
P78352_PF00595_157_C1
Q92796_PF00595_222_C1
Q13424_PF00595_79_C1
Q96RT1_PF00595_1321_C1
Q86UL8_PF00595_1144_C2
Q9UPQ7_PF00595_244_C1
Q14CM0_PF00595_75_C2
Q86UL8_PF00595_774_C1
O14936_PF00595_488_C2
Q14160_PF00595_857_C1
P78352_PF00595_309_C1
O75970_PF00595_693_C1
O75970_PF00595_1003_C1
Q8TEW0_PF00595_457_C1
Q9Y6N9_PF00595_207_C1
Q9UPQ7_PF00595_414_C1
O75970_PF00595_372_C1
Q92796_PF00595_383_C2
Q13368_PF00595_132_C1
Q61234_PF00595_80_C2
Q9UDY2_PF00595_26_C1
O75970_PF00595_1860_C2
Q9ULL8_PF00595_7_C2
Q13009_PF00595_843_C2
Q8TEW8_PF00595_380_C1
P78352_PF00595_60_C1
Q86UL8_PF00595_601_C2
Q9UDY2_PF00595_509_C1
O14640_PF00595_246_C1
Q9Y3R0_PF00595_666_C1
Q9H0A6_PF00612_189_A1
Q8IZT6_PF00612_1877_A1
Q9ULZ3_PF00619_114_B3
O43353_PF00619_438_B3
P54727_PF00627_186_C7
Q9H347_PF00627_613_C7
P54727_PF00627_361_C7
O95835_PF00627_98_C7
P61086_PF00627_158_C7
Q7Z6Z7_PF00627_1316_C7
P22681_PF00627_854_C6
Q12830_PF00628_2862_C6
Q14839_PF00628_444_C5
Q96CB8_PF00628_155_C3
mutant aa
position
scaled_gr
1.5
1.0
0.5
0.0
−0.5
−1.0
−1.5

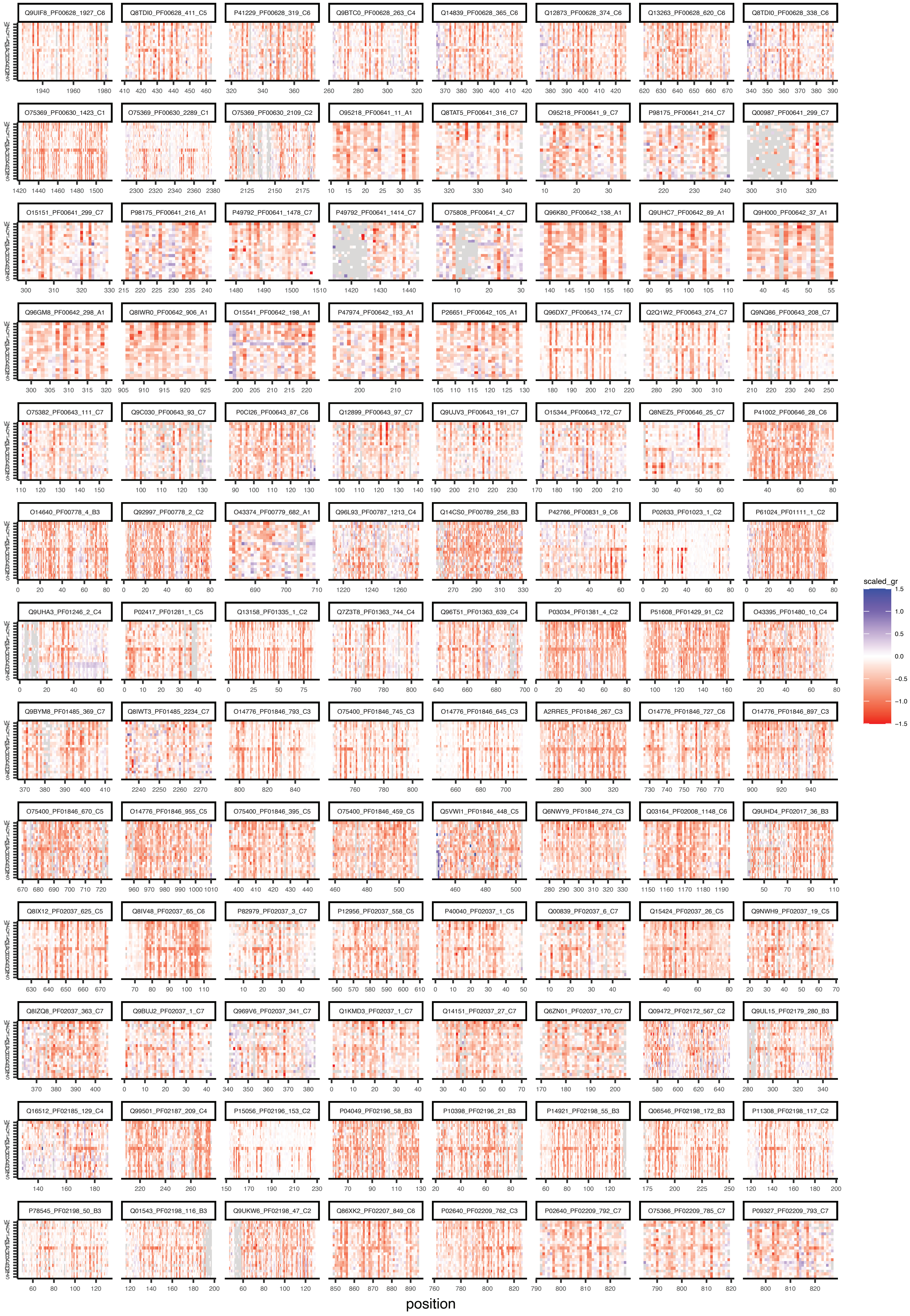
Q9UIF8_PF00628_1927_C6
Q8TDI0_PF00628_411_C5
P41229_PF00628_319_C6
Q9BTC0_PF00628_263_C4
Q14839_PF00628_365_C6
Q12873_PF00628_374_C6
Q13263_PF00628_620_C6
Q8TDI0_PF00628_338_C6
O75369_PF00630_1423_C1
O75369_PF00630_2289_C1
O75369_PF00630_2109_C2
O95218_PF00641_11_A1
Q8TAT5_PF00641_316_C7
O95218_PF00641_9_C7
P98175_PF00641_214_C7
Q00987_PF00641_299_C7
O15151_PF00641_299_C7
P98175_PF00641_216_A1
P49792_PF00641_1478_C7
P49792_PF00641_1414_C7
O75808_PF00641_4_C7
Q96K80_PF00642_138_A1
Q9UHC7_PF00642_89_A1
Q9H000_PF00642_37_A1
Q96GM8_PF00642_298_A1
Q8IWR0_PF00642_906_A1
O15541_PF00642_198_A1
P47974_PF00642_193_A1
P26651_PF00642_105_A1
Q96DX7_PF00643_174_C7
Q2Q1W2_PF00643_274_C7
Q9NQ86_PF00643_208_C7
O75382_PF00643_111_C7
Q9C030_PF00643_93_C7
P0CI26_PF00643_87_C6
Q12899_PF00643_97_C7
Q9UJV3_PF00643_191_C7
O15344_PF00643_172_C7
Q8NEZ5_PF00646_25_C7
P41002_PF00646_28_C6
O14640_PF00778_4_B3
Q92997_PF00778_2_C2
O43374_PF00779_682_A1
Q96L93_PF00787_1213_C4
Q14CS0_PF00789_256_B3
P42766_PF00831_9_C6
P02633_PF01023_1_C2
P61024_PF01111_1_C2
Q9UHA3_PF01246_2_C4
P02417_PF01281_1_C5
Q13158_PF01335_1_C2
Q7Z3T8_PF01363_744_C4
Q96T51_PF01363_639_C4
P03034_PF01381_4_C2
P51608_PF01429_91_C2
O43395_PF01480_10_C4
Q9BYM8_PF01485_369_C7
Q8IWT3_PF01485_2234_C7
O14776_PF01846_793_C3
O75400_PF01846_745_C3
O14776_PF01846_645_C3
A2RRE5_PF01846_267_C3
O14776_PF01846_727_C6
O14776_PF01846_897_C3
O75400_PF01846_670_C5
O14776_PF01846_955_C5
O75400_PF01846_395_C5
O75400_PF01846_459_C5
Q5VWI1_PF01846_448_C5
Q6NWY9_PF01846_274_C3
Q03164_PF02008_1148_C6
Q9UHD4_PF02017_36_B3
Q8IX12_PF02037_625_C5
Q8IV48_PF02037_65_C6
P82979_PF02037_3_C7
P12956_PF02037_558_C5
P40040_PF02037_1_C5
Q00839_PF02037_6_C7
Q15424_PF02037_26_C5
Q9NWH9_PF02037_19_C5
Q8IZQ8_PF02037_363_C7
Q9BUJ2_PF02037_1_C7
Q969V6_PF02037_341_C7
Q1KMD3_PF02037_1_C7
Q14151_PF02037_27_C7
Q6ZN01_PF02037_170_C7
Q09472_PF02172_567_C2
Q9UL15_PF02179_280_B3
Q16512_PF02185_129_C4
Q99501_PF02187_209_C4
P15056_PF02196_153_C2
P04049_PF02196_58_B3
P10398_PF02196_21_B3
P14921_PF02198_55_B3
Q06546_PF02198_172_B3
P11308_PF02198_117_C2
P78545_PF02198_50_B3
Q01543_PF02198_116_B3
Q9UKW6_PF02198_47_C2
Q86XK2_PF02207_849_C6
P02640_PF02209_762_C3
P02640_PF02209_792_C7
O75366_PF02209_785_C7
P09327_PF02209_793_C7
mutant aa
position
scaled_gr
1.5
1.0
0.5
0.0
−0.5
−1.0
−1.5

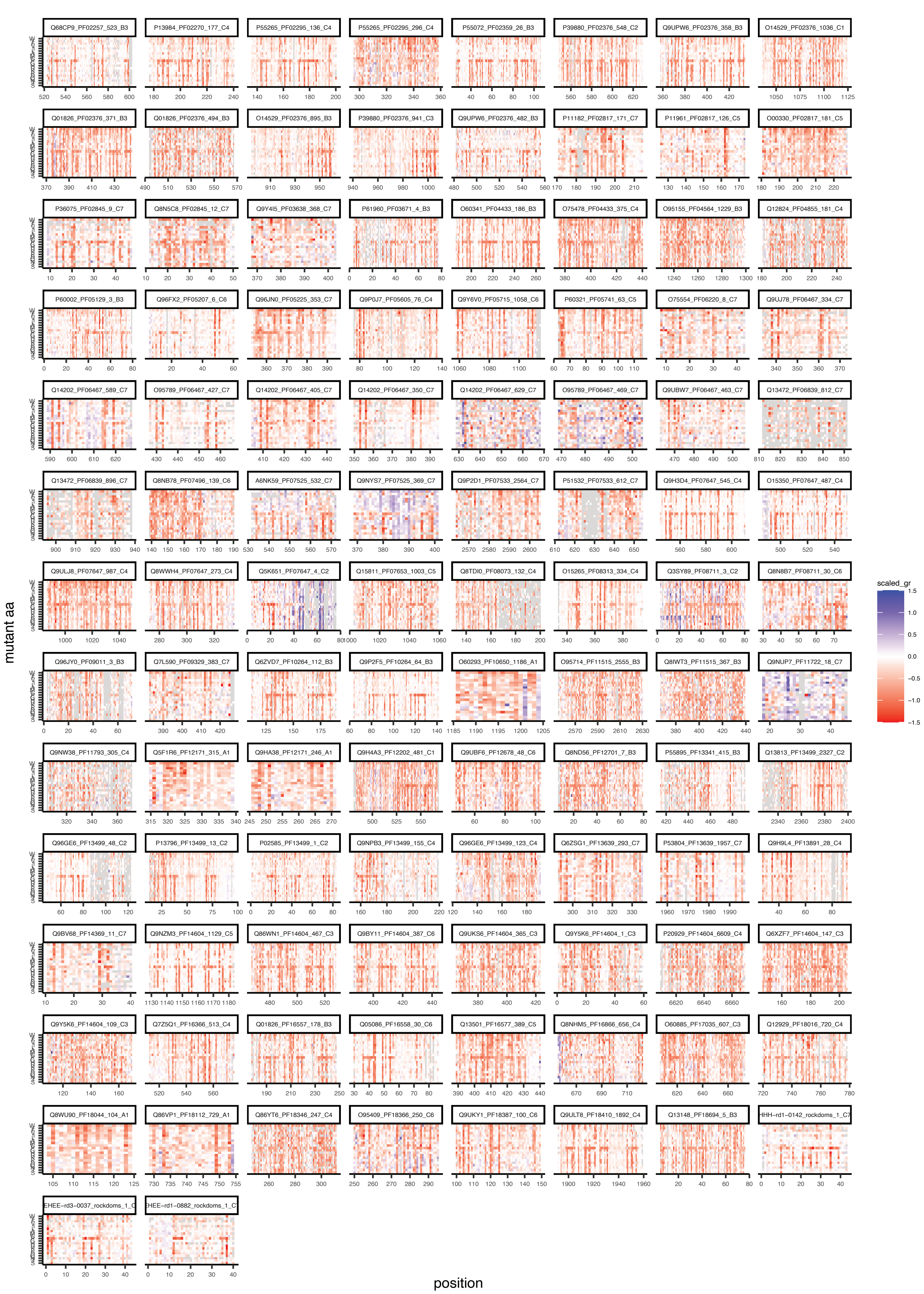
Q68CP9_PF02257_523_B3
P13984_PF02270_177_C4
P55265_PF02295_136_C4
P55265_PF02295_296_C4
P55072_PF02359_26_B3
P39880_PF02376_548_C2
Q9UPW6_PF02376_358_B3
O14529_PF02376_1036_C1
Q01826_PF02376_371_B3
Q01826_PF02376_494_B3
O14529_PF02376_895_B3
P39880_PF02376_941_C3
Q9UPW6_PF02376_482_B3
P11182_PF02817_171_C7
P11961_PF02817_126_C5
O00330_PF02817_181_C5
P36075_PF02845_9_C7
Q8N5C8_PF02845_12_C7
Q9Y4I5_PF03638_368_C7
P61960_PF03671_4_B3
O60341_PF04433_186_B3
O75478_PF04433_375_C4
O95155_PF04564_1229_B3
Q12824_PF04855_181_C4
P60002_PF05129_3_B3
Q96FX2_PF05207_6_C6
Q96JN0_PF05225_353_C7
Q9P0J7_PF05605_76_C4
Q9Y6V0_PF05715_1058_C6
P60321_PF05741_63_C5
O75554_PF06220_8_C7
Q9UJ78_PF06467_334_C7
Q14202_PF06467_589_C7
O95789_PF06467_427_C7
Q14202_PF06467_405_C7
Q14202_PF06467_350_C7
Q14202_PF06467_629_C7
O95789_PF06467_469_C7
Q9UBW7_PF06467_463_C7
Q13472_PF06839_812_C7
Q13472_PF06839_896_C7
Q8NB78_PF07496_139_C6
A6NK59_PF07525_532_C7
Q9NYS7_PF07525_369_C7
Q9P2D1_PF07533_2564_C7
P51532_PF07533_612_C7
Q9H3D4_PF07647_545_C4
O15350_PF07647_487_C4
Q9ULJ8_PF07647_987_C4
Q8WWH4_PF07647_273_C4
Q5K651_PF07647_4_C2
Q15811_PF07653_1003_C5
Q8TDI0_PF08073_132_C4
O15265_PF08313_334_C4
Q3SY89_PF08711_3_C2
Q8N8B7_PF08711_30_C6
Q96JY0_PF09011_3_B3
Q7L590_PF09329_383_C7
Q6ZVD7_PF10264_112_B3
Q9P2F5_PF10264_64_B3
O60293_PF10650_1186_A1
O95714_PF11515_2555_B3
Q8IWT3_PF11515_367_B3
Q9NUP7_PF11722_18_C7
Q9NW38_PF11793_305_C4
Q5F1R6_PF12171_315_A1
Q9HA38_PF12171_246_A1
Q9H4A3_PF12202_481_C1
Q9UBF6_PF12678_48_C6
Q8ND56_PF12701_7_B3
P55895_PF13341_415_B3
Q13813_PF13499_2327_C2
Q96GE6_PF13499_48_C2
P13796_PF13499_13_C2
P02585_PF13499_1_C2
Q9NPB3_PF13499_155_C4
Q96GE6_PF13499_123_C4
Q6ZSG1_PF13639_293_C7
P53804_PF13639_1957_C7
Q9H9L4_PF13891_28_C4
Q9BV68_PF14369_11_C7
Q9NZM3_PF14604_1129_C5
Q86WN1_PF14604_467_C3
Q9BY11_PF14604_387_C6
Q9UKS6_PF14604_365_C3
Q9Y5K6_PF14604_1_C3
P20929_PF14604_6609_C4
Q6XZF7_PF14604_147_C3
Q9Y5K6_PF14604_109_C3
Q7Z5Q1_PF16366_513_C4
Q01826_PF16557_178_B3
Q05086_PF16558_30_C6
Q13501_PF16577_389_C5
Q8NHM5_PF16866_656_C4
O60885_PF17035_607_C3
Q12929_PF18016_720_C4
Q8WU90_PF18044_104_A1
Q86VP1_PF18112_729_A1
Q86YT6_PF18346_247_C4
O95409_PF18366_250_C6
Q9UKY1_PF18387_100_C6
Q9ULT8_PF18410_1892_C4
Q13148_PF18694_5_B3
HHH−rd1−0142_rockdoms_1_C7
EHEE−rd3−0037_rockdoms_1_C
EHEE−rd1−0882_rockdoms_1_C7
mutant aa
position
scaled_gr
1.5
1.0
0.5
0.0
−0.5
−1.0
−1.5

Supplementary Tables

Supplementary Table 1: Library design: sequences, library statistics. Provided as an excel file.

Supplementary Table 2: Fitness scores and errors. Provided as an excel file.

Supplementary Table 3: Weighted mean residuals of abundance to evolutionary fitness predictions. Provided as an excel file.

Supplementary Table 4: Homolog-averaged ΔΔG predictions across families mapped to homologous domains proteome-wide. Provided as an excel file.

Supplementary Table 5: aPCA fitness scores and variant effect predictor scores. Provided as an excel file.

Supplementary Table 6: Plasmids

pGJJ162	aPCA empty	Available upon request (Material Transfer Agreement required).

Supplementary Table 7: Oligonucleotides

qPCR quantification oligos	
oGJJ152	GCCTACATACCTCGCTCTGC
oGJJ153	CAACCCGGTAAGACACGACT
Frameshifting PCR1 oligos	
oGJJ595	ACACTCTTTCCCTACACGACGCTCTTCCGATCTGCTGCTCTAGAATGGCTAGC
oGJJ595_+1	ACACTCTTTCCCTACACGACGCTCTTCCGATCTNGCTGCTCTAGAATGGCTAGC
oGJJ595_+2	ACACTCTTTCCCTACACGACGCTCTTCCGATCTNNGCTGCTCTAGAATGGCTAGC
oGJJ595_+3	ACACTCTTTCCCTACACGACGCTCTTCCGATCTHWAGCTGCTCTAGAATGGCTAGC
oGJJ595_+4	ACACTCTTTCCCTACACGACGCTCTTCCGATCTNHTAGCTGCTCTAGAATGGCTAGC
oGJJ595_+5	ACACTCTTTCCCTACACGACGCTCTTCCGATCTSSSAAGCTGCTCTAGAATGGCTAGC
oGJJ748	GTGACTGGAGTTCAGACGTGTGCTCTTCCGATCTCCCGCCACCGCCAAG
oGJJ748_+1	GTGACTGGAGTTCAGACGTGTGCTCTTCCGATCTNCCCGCCACCGCCAAG
oGJJ748_+2	GTGACTGGAGTTCAGACGTGTGCTCTTCCGATCTTNCCCGCCACCGCCAAG
oGJJ748_+3	GTGACTGGAGTTCAGACGTGTGCTCTTCCGATCTNTGCCCGCCACCGCCAAG
oGJJ748_+4	GTGACTGGAGTTCAGACGTGTGCTCTTCCGATCTGWWWCCCGCCACCGCCAAG

oGJJ748_+5	GTGACTGGAGTTCAGACGTGTGCTCTTCCGATCTACTWWCCCGCCACCGCCAAG
oGJJ748_+6	GTGACTGGAGTTCAGACGTGTGCTCTTCCGATCTCATWWWCCCGCCACCGCCAAG
oGJJ748_+7	GTGACTGGAGTTCAGACGTGTGCTCTTCCGATCTGTATADDCCCGCCACCGCCAAG
oGJJ748_+8	GTGACTGGAGTTCAGACGTGTGCTCTTCCGATCTTGGGDWWWCCCGCCACCGCCAAG

Supplementary Table 8: Sequences used in AF3 predictions

MECP2 MBD bound to methylated DNA;

- MECP2 MBD: RGPMYDDPTLPEGWTRKLKQRKSGRSAGKYDVYLINPQGKAFRSKVELIAYFEKVG DTSLDPNDFDFTVTGR
- DNA chain 1: TCTGGAA-5mC-GGAATTCTTCTA
- DNA chain 2: TAGAAGAATTC-5meC-GTTCCAGA

CRX homeodomain bound to DNA:

- CRX homeodomain: RERTTFTRSQLEELEALFAKTQYPDVYAREEVALKINLPESRVQVWFKNRRAKCRQ
- DNA chain 1: ACGTGTGCACGTGATTAGTGCCATGCAACA
- DNA chain 2: TGTTGCATGGCACTAATCACGTGCACACGT
